# Lead Concentrations in Mexican Candy: A Follow-Up Report

**DOI:** 10.5334/aogh.2754

**Published:** 2020-02-25

**Authors:** Marcela Tamayo-Ortiz, Alison P. Sanders, Maria J. Rosa, Robert O. Wright, Chitra Amarasiriwardena, Adriana Mercado-García, Ivan Pantic, Hector Lamadrid-Figueroa, Martha María Téllez-Rojo

**Affiliations:** 1 National Council of Science and Technology, Avenida Insurgentes Sur, Benito Juárez, Crédito Constructor, Ciudad de México, D.F, MX; 2 National Institute of Public Health, Universidad Colonia Santa María, Ahuacatitlán, Cerrada Los Pinos y Caminera, Cuernavaca, Morelos, MX; 3 Department of Pediatrics, Icahn School of Medicine at Mount Sinai, Madison Ave, New York, US; 4 Department of Environmental Medicine and Public Health, Icahn School of Medicine at Mount Sinai, Madison Ave, New York, US; 5 National Institute of Perinatology, Calle Montes Urales Miguel Hidalgo Lomas Virreyes, Ciudad de México, D.F, MX

## Abstract

**Background:**

Lead is a neurotoxic metal potentially affecting the developing brain. Children are particularly susceptible since they can absorb between 50% and 100% of ingested lead. There is no safe level for lead, therefore preventing exposure is crucial. We previously reported a positive association between lead concentrations found in candy and concurrent blood lead levels in Mexican children. This first report garnered media and the general public’s attention.

**Objective:**

To conduct a follow-up study to assess lead concentrations in candy brands that we previously reported with concentrations ≥0.1ppm the U.S. Food and Drug Administration’s recommended maximum lead level in candy likely to be consumed frequently by small children.

**Methods:**

In 2018 we analyzed 50 additional candy samples. Lead concentrations were analyzed by an inductively coupled plasma mass spectrometer and lead content per candy unit was calculated.

**Findings:**

We found concentrations were typically low, with a marked decrease from prior levels (2008). Nevertheless two candy units had concentrations of 0.1 ppm of lead.

**Conclusions:**

Candy may have lead concentrations up to 0.1 ppm and 1.2 μg per unit. This is a concern because candies are exported and consumed in many countries worldwide potentially resulting in human exposure. Continued public health surveillance is needed to protect populations especially vulnerable to lead exposure, especially children.

## Introduction

Preventing exposure to lead is crucial, especially in vulnerable populations such as children, who can absorb between 50% and 100% of the lead ingested in a meal or an empty stomach respectively [1]. Lead affects nearly every organ and system in the human body and is a potent neurotoxic impacting children’s neurodevelopment even at very low levels [2]. In the early 2000’s Mexican candy producers faced a controversy due to candy with elevated lead levels being exported to the US [3]. After detecting blood lead levels >10 μg/dL in Mexican migrant children (CDC, 2002), the California Public Health Department was charged with the implementation of Assembly bill 121, forbidding the importation of candy exceeding the FDA limit of 0.1 ppm of lead [4]. Our group previously published a study on the association of lead concentrations in candy and blood lead levels in children participating in the ELEMENT birth cohort in Mexico City [5]. Selected candy brands were included based on children’s report of the more frequently consumed candies. Candy samples were collected in 2008 and we reported the concentrations in 100 units. Notably we found that 6 candy units had concentrations above 0.1 ppm. Moreover, each 1 μg increment in weekly ingested lead via candy was associated with a 3% increase (95% CI: 0.1%, 5.2%) in blood lead levels after adjustment for a robust set of covariates. These results caught the interest of Mexican media resulting in a wide media coverage. In response, the Mexican Senate issued two resolutions: 1) calling the federal health authorities to implement a surveillance program for lead in food, water and consumer products and 2) to develop a blood lead monitoring program, particularly in children younger than 5 years of age and pregnant women [6]. In follow-up to our prior report, herein we report findings from re-analysis of 50 candy samples selected from brands previously reported to have lead concentrations above 0.1 ppm.

## Materials and Methods

### Lead in candy analysis

We analyzed 5 samples from each of 5 candy brands reported in our previous study with higher lead concentrations (≥0.1 ppm) that were still available (1 brand had been taken off the market). The selection of the candy in our previous study was based on a detailed candy consumption questionnaire for children, which allowed us to detect the 20 most-consumed candy reported in 2008 (the study was published in 2016) [5]. In the present study candy were purchased and analyzed in 2018 and we also included samples from: two of the candy reported among our previous study that did not have high lead concentrations, but had been reported by the FDA with high lead- namely “Miguelito” and “Pulparindo”; and three brands of lollipops- “Rockaleta Junior”, which is a variety of “Rockaleta Diablo” (0.7 ppm lead concentration in previous study), and “Bubbaloo Extreme” lollipops- for which the same brand of “Bubbaloo” chewing gum was reported in our previous study [7].

Lead concentrations in candy were analyzed in the Lautenberg Environmental Health Sciences Laboratory at the Icahn School of Medicine at Mount Sinai. Candy samples were weighed (~2 g) into a 50 mL plastic tube and digested with 2 mL of concentrated nitric acid for 24 hours and then diluted to 25 mL with deionized water. Acid-digested samples were analyzed by an inductively coupled plasma mass spectrometer (Agilent 8800 ICP-MS QQQ). Analyses were performed using an external calibration method with lutetium as the internal standard for lead. Quality control measures included analysis of the initial calibration verification standard [National Institute of Standard and Technology Standard Reference Material 1643e (trace elements in water, Gaithersburg, MD)], a 1 ng/g mixed element standard solution containing lead, continuous calibration standards, and a procedural blank. Certified Reference Material GBW 07601 was used as the quality control sample. We used a large preparation of GBW 07601 (2g /Liter) to monitor day-to-day variation. Results were given as the average of five replicate measurements. The detection limit for lead was 0.2 ng/g. Recovery of the analysis of the quality control standard by this procedure is 90%–110% with approximately 0.05 of coefficient of variation for within the day analysis. Between-assay coefficient of variation for lead was 0.03. None of the samples analyzed were below the LOD.

## Results

**Figure 1:**
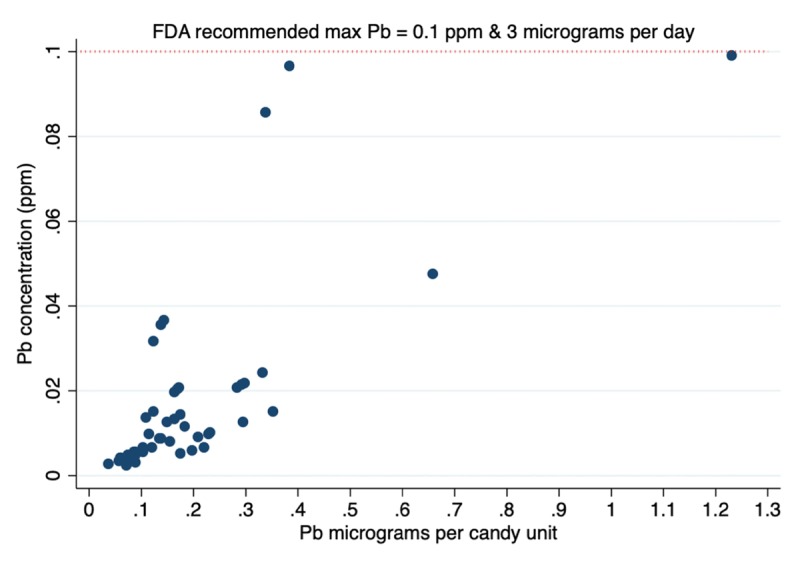
Lead concentrations (ppm) and content (micrograms) per candy unit.

None of the candy brands had a mean lead concentrations exceeding the US FDA recommended maximum level of 0.1 ppm (in Mexico there are no official regulations for lead concentrations in candy). However, 2 candy units, a *Miguelito* chili powder and a *Rockaleta Junior* lollipop had lead concentrations of 0.1 ppm. Overall our results show a marked decline in the levels of lead found in candy over the last decade (Table 1). When calculating the lead content per candy unit, none were above 3μg, which is the FDA Interim Reference Level, maximum daily intake of lead for children (Figure 1).

**Table 1:** Lead concentrations (ppm) in Candy analysed in 2008 and 2018*.

Candy Name	Arithmetic Mean (range) of 5 samples Lead Concentration (ppm)		Highest Lead Concentration (ppm) found in 1 candy piece	

	2008	2018	2008	2018

Gudupop Chile (lollipop)	0.096 (0.059–0.157)	0.018 (0.013–0.021)	0.16	0.02
Indy Marimbas (gummy)	0.219**	0.004 (0.002–0.006)	0.22	0.01
Miguelito (chili powder)	0.047 (0.040–0.058)	0.057 (0.031–0.096)	0.06	0.10
Ricaleta Chamoy (lollipop)	0.192**	0.006 (0.004–0.008)	0.19	0.01
Rockaleta Diablo (lollipop)	0.700**	0.011 (0.009–0.015)	0.70	0.01
Tutsi Pop (lollipop)	0.032 (0.005–0.129)	0.004 (0.003–0.004)	0.13	0.00
Pulparindo (Tamarind candy)	0.008 (0.006–0.010)	0.027 (0.020–0.047)	0.01	0.05
Bubbaloo Xtreme blueberry (lollipop)	Not analyzed	0.005 (0.002–0.011)	Not analyzed	0.01
Bubbaloo Xtreme strawberry (lollipop)	Not analyzed	0.006 (0.004–0.008)	Not analyzed	0.01
Rockaleta Junior (lollipop)	Not analyzed	0.030 (0.009–0.099)	Not analyzed	0.10

* Highlighted concentrations were ≥0.1 ppm, the US- FDA permissible lead limit. ** Only 1 candy sample analyzed in 2008.

## Discussion

Overall, the results of our analyses are encouraging in terms of consumer safety, albeit our sample size was small and two candy units (4% of the samples) had concentrations at the upper limit of recommendation. Nonetheless, if lead levels in candy exceeds the recommended level, even by a small amount, this could represent a source of lead exposure that is particularly important among young children. Continued monitoring would be relatively inexpensive yet critically important for public health surveillance. In our initial study, children aged 2 to 6 years reported eating almost 3 candies per day, with some eating up to 32 candies per day. As shown in Figure 1, at least one of the candy units analyzed had a Pb content above 1 μg. With the wide variability in candy consumption, potential concern for lead toxicity exists for children ingesting moderate to high quantities of candy, despite lead concentrations of individual candies being below regulated levels. It is important to highlight there is no “safe” level of lead for children, and repeated exposure, even to low doses can affect short and long term health [8,9].

Children and pregnant women, who are particularly vulnerable to the effects of lead exposure, may consume candy and most likely not a single unit (especially children). According to the results of the 2012 Mexican Nutrition and Health Survey, sweets accounted for ~38% of total intake of added sugars for toddlers and ~30% for preschoolers [10]. A study evaluating lead concentrations in Nigerian candy, found lead concentrations >0.1 ppm of lead in 80% of the 50 samples analyzed [11]. Today, it is likely consumers around the world can find imported candy, hence the need for a stricter lead-free candy production.

A key question that remains to be studied is the source of lead in the candy. We posit that three potential primary contributing sources are the 1) ingredients, 2) container or cooking equipment, or 3) wrapper [3]. In a detailed report, the Orange County Register-Excelsior from California pointed at powdered chili pepper as the main source of lead without a clear cause of how it contains lead – if it is by biologic uptake or somehow added in its production process [7]. Nonetheless other candy and food items not containing chili such as chocolates or turmeric have shown high lead levels (> 0.1 ppm) [12,13]. In our previous study, one of the non-chili candies, a “Tutsi-pop lollipop” had a lead concentrations of 0.1 ppm (see Table 1). If lead is found in the raw materials for the candy, it poses a challenge to producers having to certify their providers. In the early 1990’s high lead concentrations were detected in Mexican candy wrappers and ceramic containers [14]. In 1993 the Mexican Health authorities released a regulation limiting the use of lead in ink [15]. In response, candy producers with large exports implemented stricter quality controls and lead was no longer detected in the wrappers. However, a well-known source of lead exposure in Mexico is the use of traditional lead-glazed low-temperature ceramics to prepare, serve and store food [16]. Lead will leach into food that is in contact with the surface of these ceramics, particularly acidic food such as tamarind candy. This type of ceramic is widely used in Mexico and is linked to cultural identity [16]. Still today, artisans lack access to high temperature kilns or adequate lead-free glazes to make a change to producing safer ceramics economically feasible, rendering a complex public health challenge in Mexico. An additional unexplored type of candy that could potentially contain lead are products sold as “traditional Mexican candies” – such as candied fruits, caramelized amaranth and peanut bars which may be prepared in lead-glazed ceramics. These are popularly consumed candy and commercialized by weight in markets without a label or an identifiable producer. While the source of lead in candies was not the objective of our present study, clearly more work must be done to avoid lead reaching consumer products.

Consumers worldwide are entitled to demand a regular surveillance program for lead in consumer products and timely risk communication, from the health authorities. A good example is the California’s Department of Public Health, which tests candy periodically and publicly publishes their results online [17]. In Mexico, the regulation (NOM-131-SSA1-2012) specifies a limit for lead in consumer products of 0.2 ppm for children less than 3 years old [18]. The Federal Commission for Protection of Sanitary Risks (COFEPRIS) is tasked with monitoring lead in consumer products, however there is no program that provides publicly available results. Our study does not intend to replace a possible surveillance program. The candy reported in this study is only a small fraction of the variety of candy available in the Mexican Market and the selection of the candy brands did not follow a methodology intended to be representative of the Mexican candy market. The inclusion of candy in this study was based on the results from candy with high lead levels in our previous study. The candy brands included are mostly from major and mid-size brands: Sonrics, Tutsi Pop, De la Rosa, Miguelito, Productos Yaukos and Indy; in 2009 in Mexico there were 1,220 candy producers, 201 chocolate producers, and 29 chewing gum producers reported, therefore an study including an exhaustive sample from all producers would be very complex [19].

In addition to the lack of information on lead levels in food, national representative data of blood lead levels in the Mexican population have only recently become available, although they include only children between 1–4 years old, in vulnerable populations (<100,000 inhabitants). The prevalence of blood lead levels ≥5μg/dL (the current blood lead level limit established by the Mexican Health System, and in accordance to the U.S. CDC) was 21.8% and there was a strong association between the use of lead-glazed ceramics and higher blood lead levels [20,21]. Results from epidemiologic studies have shown that overall and across the years, the percentages of Mexican children younger than 5 years old with blood lead concentrations ≥5μg/dL range from 8% - 15% and average levels in both children and pregnant women remain nearly 3 times higher than those estimated in the US during the same years [16,22,23]. A targeted monitoring system of blood lead concentrations representative of the Mexican population, similar to the National Health and Nutrition Examination Study (NHANES) in the U.S, would begin to provide information needed to understand the need for national prevention of lead exposure across all age groups but especially in vulnerable populations like children and pregnant women.

## Conclusion

We found that lead concentrations in selected Mexican candies were typically low, with a marked decrease from prior levels. Yet some lead levels in Mexican candy still exceed the recommended level. Candy-based exposures, even in trace amounts, could represent a source of lead exposure that is particularly important among young children.
